# Metabolomics-Based Clinical Efficacy of Compound Shenlu Granule, a Chinese Patent Medicine, in the Supportive Management of Aplastic Anemia Patients: A Randomized Controlled Pilot Trial

**DOI:** 10.1155/2021/6655848

**Published:** 2021-09-30

**Authors:** Zhou Feng, Xiaoying Hu, Weiying Qu, Xiaoqin Zhu, Jiaying Lu, Zhongdi Huang, Lin Zhao, Pei Chen

**Affiliations:** Department of Hematology, Shuguang Hospital Affiliated to Shanghai University of Traditional Chinese Medicine, Shanghai 201203, China

## Abstract

**Objective:**

To explore the clinical efficacy and mechanism of compound Shenlu granule (SLG) treatment in patients with aplastic anemia (AA).

**Methods:**

A total of 89 AA patients were randomly divided into an SLG supportive group (group A, *n* = 44) and a control group (group B, *n* = 45) while continuing Western medical management. After 6 months, hemograms, traditional Chinese medicine (TCM) syndrome scores, and overall clinical efficacy rate were assessed. Serum metabolomics characteristics were observed using ultraperformance liquid chromatography-mass spectrometry after SLG intervention.

**Results:**

The levels of red blood cell (RBC), hemoglobin (Hb), and platelet (PLT) were increased in both groups after treatment for 6 months (*P* < 0.05), and in group A, the elevation of PLT became much more significant (*P* < 0.01). The TCM syndrome score was lower in group A than in group B after treatment (*P* < 0.05). Metabolomics data showed a significant difference in the patients using SLG after 6 months, and 14 biomarkers were identified.

**Conclusion:**

SLG supportive treatment showed positive results in patients with AA, and metabolomics data indicated that SLG influenced aminoacyl-tRNA biosynthesis and glycerophospholipid metabolism to gradually return to normal.

## 1. Introduction

Aplastic anemia (AA) is a rare and heterogeneous disease with 2-3-fold higher annual incidence rates in Asian countries [1]. The disease is characterized by pancytopenia and hypocellular bone marrow. Blood counts of various types can be decreased drastically, thus making the patients more vulnerable to infection [2].

Over the last 25 years, significant improvement has been made in the treatment of AA, such as allogeneic stem cell transplant and immunosuppressive therapy [3]. However, because of multiple comorbidities or expensive medical cost, large numbers of patients tend to seek supportive treatment with an emphasis on ameliorating their hematological profile, reducing the complications, and improving their quality of life [4].

Traditional Chinese medicine (TCM) has been utilized for more than 200 years in the treatment of various diseases and regarded as a form of “complementary and alternative medicine” in Chinese healthcare system. In the Chinese literature, TCM has been shown to significantly slow down the progression of AA and improve the quality of life among AA patients [5, 6]. Compound Shenlu granule (SLG) is a classical hospital preparation for treating AA due to the kidney yang deficiency pattern, which has been used in clinical practice for more than a decade in Shanghai Shuguang Hospital. Previous studies have reported that SLG has shown encouraging improvement in AA patients. Evidence suggests that SLG may regulate the immune system and adjust the level of cytokines and the ratio of Treg cells; however, its therapeutic material basis and mechanisms are still undefined [7, 8].

Metabolomics is a powerful analysis method to evaluate the characteristics and interactions of metabolic components during a specific period [9]. By investigating changes in metabolomics, we can identify biomarkers and biological pathways when the biological system is stimulated or disturbed and thus understand the action of diseases and drugs [10].

In this study, we carried out a pilot clinical trial on AA patients to evaluate the clinical efficacy of SLG supportive treatment. Moreover, a metabolomics screening study combined with pattern recognition methods was performed to explore the regulatory mechanisms of SLG.

## 2. Methods and Materials

### 2.1. Inclusion Criteria

The inclusion criteria were as follows: (a) patients who meet the AA diagnostic standard of mainstream medicine [11] and kidney yang deficiency Chinese medicine pattern identification [12]; (b) patients with no other serious diseases (e.g., cardiovascular diseases, severe hepatic and renal dysfunction, severe infection, and mental diseases); (c) patients aged 15–80 years; and (d) patients participated in the study voluntarily and signed informed consent.

### 2.2. Exclusion Criteria

The exclusion criteria were as follows: (a) patients who were pregnant or breastfeeding; (b) patients diagnosed with other hematological illnesses or malignancies; (c) patients diagnosed with complex TCM syndrome; and (d) patients failed to take their medication as prescribed.

### 2.3. General Data

Between March 2017 and March 2019, 89 patients from Shanghai Shuguang Hospital were recruited into this study. Simple randomization was adopted according to a computer-generated random number sequence. All patients were randomly divided into the SLG treatment group (group A, *n* = 44) and the control group (group B, *n* = 45). This study followed the Declaration of Helsinki and was approved by the Institution Committee of Ethics of Shanghai Shuguang Hospital (2020-sgys-028). Written informed consent was signed by all participants.

### 2.4. Therapies

All enrolled patients received mainstream medicine treatment protocols, including stanozolol (4–6 mg·d^−1^·tid) and cyclosporine A (3–6 mg·kg^−1^·d^−1^, with the serum concentration maintained at 150–250 ng/L). On the basis of mainstream medicine maintenance, patients in group A were treated with compound Shenlu granule, which comprised the following prepared herbs: Hongshenxu (*red ginseng tails*), Lujiao (*Colla Cornus Cervi*), Fuzi (*Radix Aconiti Lateralis Preparata*), Rougui (*Cortex Cinnamomi Cassiae*), Tusizi (*Semen Cuscutae*), Dihuang (*Radix Rehmanniae*), Zhiguiban (*processed tortoise shell*), Buguzhi (*Fructus Psoraleae*), Gouqizi (Fructus Lycii), and Roucongrong (*Cistanche deserticola*). The TCM herbs were imported in bulk from a regular source in Shanghai and were made into granules by the TCM center of Shanghai Shuguang Hospital (lot number: 200202). The outpatient dosage was 12g per dose, three times a day with warm water delivery. The treatment period for both groups lasted 6 months.

### 2.5. Outcomes

#### 2.5.1. Common Index

Gender, age, body weight and height, and history of present and past illness were recorded. After 6 months of medication, fasting blood was collected and stored in a refrigerator (−80°C) for further analysis after isolating serum.

#### 2.5.2. Curative Effect Index

(a) Hemogram: patients were checked routinely for blood every month. (b) TCM syndrome assessment: the TCM syndrome scores were assessed according to the Scheme of Clinical Diagnosis and Treatment in 2010.

#### 2.5.3. Efficacy Evaluation Criteria

Criteria in mainstream medicine are as follows. (a) Basically cured: anemia and bleeding disappeared, with a hemoglobin level of 120 g/L in male and 110 g/L in female patients, without recovery within 1 year. (b) Remission: anemia and bleeding disappeared, with a hemoglobin level of 120 g/L in male patients and 110 g/L in female patients. The white blood cell (WBC) count reached 3.5 × 10^9^/L, and the platelet count demonstrated an improvement. Disease remained stable or improved within 3 months. (c) Improved: anemia and bleeding improved significantly in a transfusion-independent manner, and hemoglobin increased above 30 g/L. Disease stays stable for more than 3 months. All three criteria should be assessed in the condition of no transfusion in the last 3 months. (d) Invalid: symptoms and hemogram were not improved significantly after therapy. Disease control rate was defined by the total rate of basically cured, remission, and improved patients. Criteria in TCM: score improving rate = (score before treatment − score after treatment)/score before treatment × 100%.

### 2.6. Sample Preparation for Metabolomics

After 6 months of treatment, the serum of patients in the two groups was removed from the −80°C temperature refrigerator and frozen at 4°C. Two hundred microliters of serum was precisely transferred into a 1.5 mL centrifuge tube, and 600 *μ*L of cold methanol was dipped into it. The tube was mixed for 30 s and centrifuged for 10 min at 14,000 rpm, and a sample solution was prepared. Quality control (QC) samples were prepared by mixing 20 *μ*L of serum from each sample and extracted using the same procedures as the test samples. Two QC samples were inserted regularly for every 10 samples.

### 2.7. UPLC-MS Analysis

UPLC-MS was performed on a Thermo Accela 1250 UPLC/Q-Exactive system (Thermo Fisher Scientific, San Jose, CA, USA) equipped with a binary solvent delivery pump. Hypersil GOLD C18 column (150 mm*∗*2.1 mm i.d., 1.9 *μ*m; Thermo Fisher Scientific) was used. Solvent: the column was maintained at 40°C, and separation was achieved using the following gradient: 0–2% B, 0–0.5 min; 2–30% B, 0.5–5 min; and 30–98% B, 5–10 min; the composition was held at 98% B for 6 min and then returned to 2% B. The flow rate was set at 0.30 mL/min, where B is acetonitrile (0.1% formic acid) and A is aqueous formic acid (0.1% formic acid). Injection volume was 2 *μ*L. The mass spectrometric data were collected using an electrospray ionization (ESI) source operating in either positive or negative ion mode in the range of 70–1050 *m*/*z*. The collision energy was set from 15 to 40 eV (−15 to −40 eV) for each run.

### 2.8. Statistical Analysis

Statistical analyses were performed with SPSS 20.0. Data are expressed as the mean ± standard error (SE). Continuous variables that were not normally distributed were analyzed using the nonparametric Mann–Whitney *U* test. Normally distributed continuous variables were analyzed using unpaired Student's *t* test. Categorical variables were compared using the chi-square test. Two-sided *P* values of less than 0.05 were considered statistically significant.

The raw metabolomics data were analyzed by Compound Discoverer 2.0 software (Thermo Fisher Scientific, San Jose, CA, USA). After RT alignment, peak discrimination, ion filtering, and identification, a feature file was obtained with a three-dimensional data table including *m*/*z*, RT, and intensities, and *m*/*z*-RT pairs were used as the identifier for each feature. Extracted matrices that were shown at 80% or above in each group were constant, or they were identified to be removed. The positive mode and negative mode data were combined to form a combined data set and then imported into SIMCA 14.0 software (Umetrics, Umeå, Sweden). After mean centering and unit variance scaling, principal component analysis (PCA) and orthogonal partial least-squares-discriminant analysis (OPLS-DA) were performed to analyze the metabolic alterations. Variable importance in the projection (VIP) >1.0 was considered relevant for discrimination. KEGG (http://www.kegg.jp/) and MetaboAnalyst 3.0 (http://www.metaboanalyst.ca/) were used to identify the metabolic pathways.

## 3. Results

### 3.1. Features of Patients

During the trial, 9 of 89 patients were withdrawn: five in group A and four in group B. Patients in group A (*n* = 39) were aged 41.08 ± 2.05 years (mean ± SE) and included 19 men and 20 women. Patients in group B (*n* = 41) were aged 40.24 ± 2.19 years and included 23 men and 18 women. There was no significant difference in general data between the two groups (*P* > 0.05) (Table 1).

### 3.2. Comparison of Hemograms

There were no differences in the hemograms of patients in the groups before treatment (*P* > 0.05). After 6 months, the WBC counts increased significantly in group A, from 3.31 ± 0.25 × 10^9^/L to 4.32 ± 0.21 × 10^9^/L (*P*=0.002), whereas in group B, no significant difference was found (*P*=0.153). The levels of RBC and Hb were increased in both groups (*P* < 0.05), and only in group A, the increase of PLT become significant (*P*=0.041) (Table 2).

### 3.3. Comparison of TCM Syndrome Score and Therapeutic Effect

**Figure 1:**
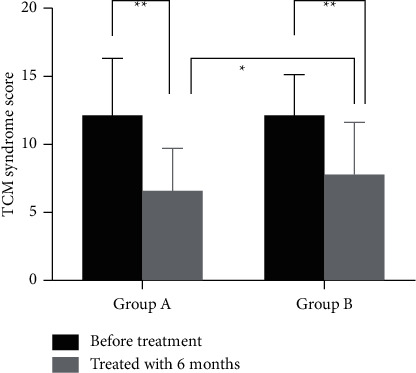
TCM syndrome scores of the two groups evaluated before and after treatment (^*∗*^*P* < 0.05;  ^*∗∗*^*P* < 0.01).

No differences were observed in the TCM syndrome score of the two groups before treatment (12.2 ± 0.69 and 11.9 ± 0.51, *P*=0.723). After treatment for 6 months, both groups showed improvement in TCM syndrome, and syndrome scores decreased significantly (6.4 ± 0.50 and 7.9 ± 0.60, *P* < 0.01). The TCM syndrome score was lower in group A than in group B after treatment (Figure 1; *P*=0.046). Out of the patients who completed the study, disease control rate of group A was 79.49% and that of group B was 68.29% (*P* < 0.05).

### 3.4. Serum Metabolites Identification

**Figure 2:**
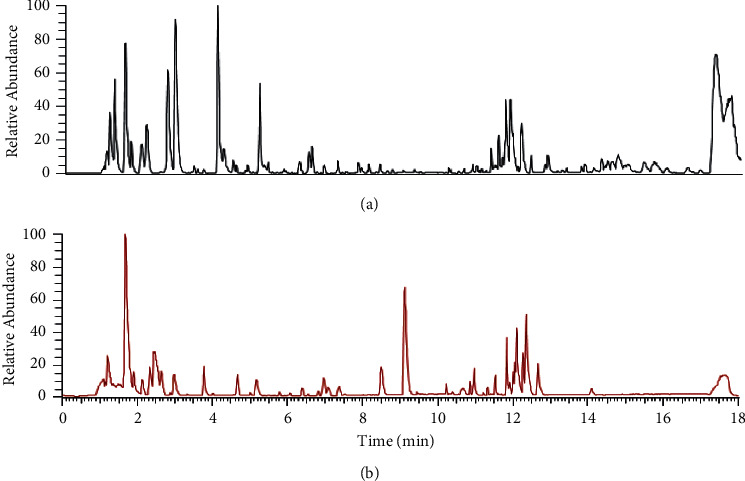
Representative chromatogram of plasma samples of the SLG group derived from UPLC-Q-Exactive. (a) ESI positive mode and (b) ESI negative mode.

**Figure 3:**
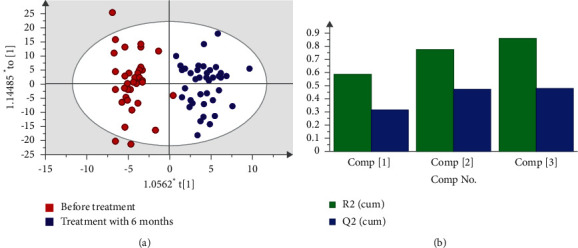
OPLS-DA scores and model parameters before and after treatment in group B. (a) Score scatter plot and (b) the model parameters (*R*^2^*X* = 0.22, *R*^2^*Y* = 0.87, and *Q*^2^ = 0.49).

A total of 2213 peaks were detected by UPLC-MS from 80 serum samples (Figure 2). Pooled quality control samples clustered tightly in principal component analysis models, indicating excellent repeatability. To determine whether TCM affected the metabolic pattern and to identify the metabolites with significant changes, OPLS-DA was used. Scoring plots generated from OPLS-DA models revealed a clear separation between the two groups (Figure 3(a)). The OPLS-DA model showed an *R*^2^*X* value of 0.22, an *R*^2^*Y* value of 0.87, and a *Q*^2^ (cum) value of 0.491 (Figure 3(a)). Fourfold cross-validations of *R*^2^*Y* and *Q*^2^ indicated good fitness and predictability, and the negative *Q*^2^ from 999 permutation tests suggested no overfitting in the OPLS-DA models. The results showed that there were 16 positive and 43 negative ion modes of distinct metabolites in the two groups after 6 months of treatment, among which 14 metabolites, including amino acids, sphingolipids, glycerophospholipids, and other endogenous compounds, could be identified (Table 3).

**Figure 4:**
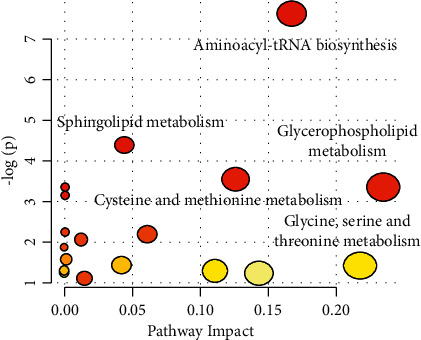
The main metabolic pathways based on potential biomarkers.

Based on 14 potential biomarkers between the two groups, pathway analysis based on MetaboAnalyst 3.0 was carried out. As shown in Figure 4, the potential target metabolic pathways were aminoacyl-tRNA biosynthesis, glycerophospholipid metabolism, linoleic acid metabolism, and sphingolipid metabolism (*P* < 0.05).

### 3.5. Adverse Reactions

The most common side effect reported was mild gastrointestinal symptoms such as bloating, loose stools, and constipation. Nausea was reported by 2 patients. All symptoms resolved after adequate direction for drug use. There were no changes observed in renal or liver function when compared to their baseline levels.

## 4. Discussion

The combined treatment of TCM and WM is a common practice in China and adopted routinely in the clinical treatment of AA [4]. In this study, we found that SLG treatments based on a kidney reinforcing pattern added to their ongoing supportive management resulted in a higher disease control rate, which is consistent with previous publications in China [13, 14]. Metabolomic analysis showed that intake of SLG in AA patients brought about significant biochemical changes. The effect of SLG mainly influenced aminoacyl-tRNA biosynthesis and glycerophospholipid metabolism in AA patients, indicating that SLG treatment gradually drove amino acid and lipid metabolism in patients who returned to normal.

According to the principle of TCM, the main roles of the kidney are to store essence, dominate development and reproduction, control bone, and nourish marrow [15]. The essence and blood have basically the same source, while the essence can transform into blood. Kidney essence is the origin of bone marrow. The formula of compound Shenlu granules was designed based on traditional Chinese medicine theory, and many of the herbs in the formulation were classified as “nourishing kidney and blood.” Modern biomedical science has revealed that red ginseng and Lujiaojiao can promote hemopoiesis by multiple mechanisms, including proliferation of colony-forming unit progenitors of hemopoietic cells [16], inhibition of inflammatory cytokines [17, 18], suppression of apoptosis, and disturbance of aberrant signaling pathways [19]. Some of the component herbs in SLG are also reported to have immune-regulatory activities (wolfberry fruit, turtle shell, grilled *Psoralea* fruit, monkshood, Semen Cuscutae, cinnamon, etc.) or hemopoiesis-promoting activities (*Rehmannia* root, *Cistanche deserticola*, etc.) [14, 20–22].

The prescription of traditional Chinese medicine was often based on TCM-type diagnostic of the patient's syndrome, which is a practice inherent in TCM theory of personalized treatment. It has been found that there is a relationship between TCM types of AA and immune function [23]. Based on the kidney reinforcing pattern, we found that the recovery of hemograms in the SLG combined treatment group was superior to the therapeutic effect in the mainstream medicine alone group. The improvement in WBC counts and platelet recovery was greater in the TCM group, and the TCM syndrome scores were decreased significantly after 6 months compared with the mainstream medicine alone group. Similar results were obtained for the treatment of myelodysplastic syndrome, myelofibrosis, and thalassemia with TCM concoctions [14].

Metabolomics can play a positive role in the disease control rate evaluation and clarification of the mechanism of TCM [24]. Our metabolomics study showed that there were significant differences in amino acid and lipid metabolism after herbal treatment. The metabolites related to amino acids and lipid synthesis have been associated with immune function and the cell membrane, which may ultimately influence the therapeutic effect.

Tryptophan metabolism was one of the changed pathways identified in the patients treated with SLG. Tryptophan can be involved in different pathways, including the kynurenine pathway, which has been reported to be regulated by altered immune responses [25]. Lower levels of tryptophan are often related to an increased oxidative stress response and various diseases, including cancer, neurodegenerative disorders, and aplastic anemia [26]. The metabolite indole-3-acetamide, which was identified from another tryptophan metabolism pathway, was also observed to be regulated by TCM treatment [27]. Our findings suggested that SLG practice resulted in the improvement of tryptophan metabolism in AA patients. Given the mediating effect of tryptophan metabolism in immune systems, metabolite disorders could be regulated.

Glycerophospholipid metabolism regulation was also identified in the SLG combined treatment group, with PC upregulated and elevated significantly (VIP > 1 and *P* < 0.05). PC is the largest glycerophospholipid in mammals. They provide the major structural constituents in biomembranes and membrane lipids within cells. Changes in membrane chemical composition and metabolic disorders in certain types of cells have been observed in AA patients [28]. In this study, SLG had a strong impact on glycerophospholipids, which may have the effect of maintaining the normal function of cells and enhancing lipid metabolism, thus stabilizing the cell membrane.

Phenylalanine metabolism was also identified in the SLG combined treatment group. Phenylalanine, which is one of the essential aromatic amino acids to produce various proteins, has been used as an oxidative stress biomarker [29]. Moreover, it can be translated into tyrosine in body tissues such as the liver. Previous studies have shown that immunity, abnormal liver and kidney function, nervous system diseases, inheritance, and other factors were significantly correlated with phenylalanine metabolic disorder [30, 31]. Maintaining normal metabolism of phenylalanine could help to ensure the body's growth, development, and physiology. In this study, the results showed that the phenylalanine content in the SLG group increased in the blood, which may suggest that immune function was improved by affecting the phenylalanine metabolic pathways.

This was a pilot clinical trial to probe the clinical efficacy and effect of SLG combined therapy on endogenous metabolites for AA. The sample size in the study was relatively small. Larger samples with long-term studies are necessary to eliminate the difference in genetic background, and if uniform diets and living habits could be taken into control, the results will be more meaningful. Based on this study, a long-term clinical trial will be carried out by our team to evaluate the safety and efficacy of SLG for AA in the near future.

## 5. Conclusions

In this study, the clinical trial indicated that a combination of SLG and WM therapies has a positive role in AA treatment. Based on kidney reinforcing syndrome, the hemocyte profile in the SLG combined treatment group was superior to the therapeutic effect in the WM alone group, and the TCM syndrome scores were decreased significantly after 6 months. A metabolomics study based on UPLC-MS analysis illustrated that SLG influenced amino acid and glycerophospholipid metabolism, which may provide new insights into the potential mechanism.

## Figures and Tables

**Table 1:** Comparison of clinical efficacy of two groups (*n* (%)).

	Basically cured	Remission	Improved	Invalid	Disease control rate
Group A (*n* = 39)	3 (7.69)	19 (48.72)	9 (23.08)	8 (20.51)	79.49^*∗*^
Group B (*n* = 41)	3 (7.32)	7 (17.07)	18 (43.90)	13 (31.71)	68.29

^*∗*^ *P*=0.007; *χ*^2^ = 7.36.

**Table 2:** Comparison of hemograms between two groups.

	Time	WBC (10^9^/L)	RBC	Hb (g/L)	PLT (10^9^/L)
Group A	Baseline	3.31 (0.25)	2.49 (0.13)	87.90 (4.16)	31.61 (4.83)
	After treatment	4.32 (0.21)^*∗∗*^	3.01 (0.14)^*∗∗*^	109.30 (4.63)^*∗∗*^	49.13 (7.05)^*∗*^
	*t* value	3.20	2.73	3.48	2.09
	*P* value	0.002	0.008	0.001	0.041
Group B	Baseline	3.40 (0.19)	2.31 (0.09)	81.20 (3.89)	34.33 (4.43)
	After treatment	3.89 (0.28)	2.81 (0.10)^*∗∗*^	96.76 (3.58)^*∗∗*^	44.13 (5.26)
	*t* value	1.446	3.71	2.95	1.423
	*P* value	0.153	<0.000	0.004	0.159

WBC, white blood cell; RBC, red blood cell; Hb, hemoglobin; PLT, platelet. ^*∗*^*P* < 0.05;  ^*∗∗*^*P* < 0.01.

**Table 3:** Identification results of potential biomarkers after SLG intervention.

Name	HMDB	Formula	Trend	VIP
Tryptophan	HMDB00929	C_11_H_12_N_2_O_2_	Up	1.21
Glutamate	HMDB00161	C_5_H_9_NO_4_	Up	1.39
Serine	HMDB00187	C_3_H_7_NO_3_	Up	1.15
Methionine	HMDB00696	C_5_H_11_NO_2_S	Up	1.42
Ornithine	HMDB0000214	C_5_H_12_N_2_O_2_	Up	2.03
PA 16:0	HMDB00674	C_39_H_73_O_8_P	Up	2.13
PC 22:5	HMDB08680	C_48_H_84_NO_8_P	Up	1.70
PC 14:0	HMDB07867	C_37_H_69_O_8_P	Up	1.61
Propionylcarnitine	HMDB00824	C_10_H_20_ClNO_4_	Up	2.85
Palmitic acid	HMDB00220	C_19_H_38_O_4_	Up	2.17
Acetylspermidine	HMDB01276	C_9_H_21_N_3_O	Down	1.06
Sphingosine	HMDB00252	C_18_H_37_NO_2_	Down	1.47
5-HETE	HMDB11134	C_20_H_32_O_3_	Down	1.08
Azelaic acid	HMDB00784	C_9_H_16_O_4_	Down	1.35

## Data Availability

The data used to support the findings of this study are available from the corresponding author upon reasonable request.

## Abbreviations

AA: Aplastic anemia
ESI: Electrospray ionization
HGB: Hemoglobin
OPLS-DA: Orthogonal partial least-squares-discriminant analysis
PLT: Platelet
PCA: Principle component analysis
QC: Quality control
RBC: Red blood cell
RT: Retention time
SLG: Compound Shenlu granule
TCM: Traditional Chinese medicine
UPLC-MS: Ultraperformance liquid chromatography-mass spectrometry
VIP: Variable importance in the projection
WBC: White blood cell
WM: Western medicine.

## Supplementary Materials

Supplementary Materials Figure S1: line plots of quality control (QC) samples generated by PCA using components 1 and 2. Peak area deviation could be evaluated by distribution of the runs (*X*-axis: run order; *Y*-axis: standard deviation). (a) QC plot for the first component from LC-(+) ESI-MS data, (b) QC plot for the second component from LC-(+) ESI-MS data, (c) QC plot for the first component from LC-(ESI)-MS data, and (d) QC plot for the second component from LC-(ESI)-MS data. Table S1: information of potential biomarkers after SLG intervention.
