# Supplementary material for: Metabolomics-Based Clinical Efficacy of Compound Shenlu Granule, a Chinese Patent Medicine, in the Supportive Management of Aplastic Anemia Patients: A Randomized Controlled Pilot Trial
Source: Evid Based Complement Alternat Med. 2021 Sep 30;2021:6655848. doi: 10.1155/2021/6655848 (PMC8497100; doi:10.1155/2021/6655848)
Supplement: Supplementary Materials — Figure S1: line plots of quality control (QC) samples generated by PCA using components 1 and 2. Peak area deviation could be evaluated by distribution of the runs (X-axis: run order; Y-axis: standard deviation). (a) QC plot for the first component from LC-(+) ESI-MS data, (b) QC plot for the second component from LC-(+) ESI-MS data, (c) QC plot for the first component from LC-(ESI)-MS data, and (d) QC plot for the second component from LC-(ESI)-MS data. Table S1: information of potential biomarkers after SLG intervention. [file 6655848.f1.docx]

**Supporting Information**

**Metabolomics-Based Clinical Efficacy of Compound Shenlu Granules, a Chinese Patent Medicine in the supportive management of Aplastic Anemia patients**

Zhou Feng^#^, Xiaoying Hu^#^, Weiying Qu, Xiaoqin Zhu, Jiaying Lu, Zhongdi Huang, Pei Chen^*^

From Department of Hematology，Shuguang Hospital Affiliated to Shanghai University of Traditional Chinese Medicine，Shanghai 201203，China.

^#^These authors contributed equally.

^*^Corresponding author.

Pei Chen, Email: chenpei3020@163.com.


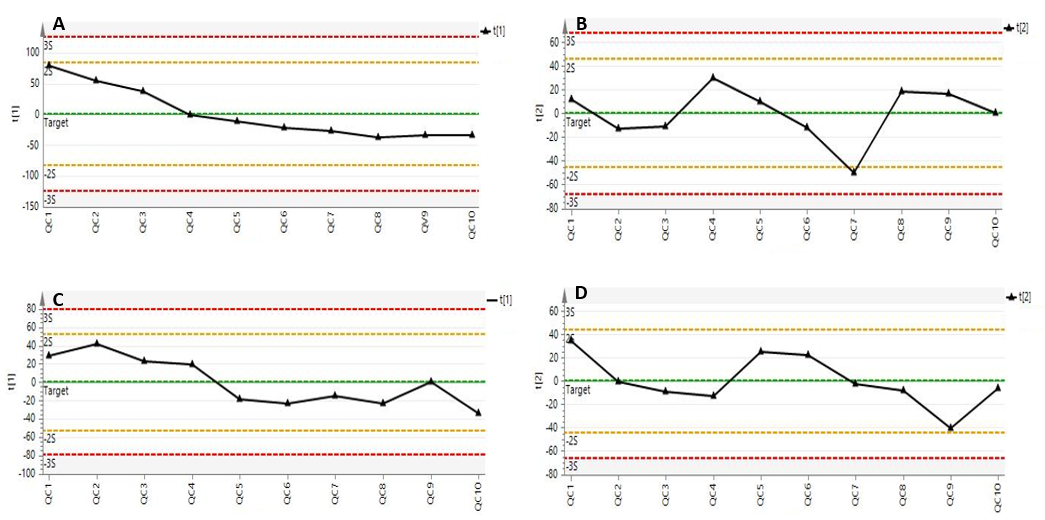
**Figure S1 Line plots of quality control (QC) samples generated by PCA using component 1 and 2. Peak area deviation could be evaluated by distribution of the runs. X-axis: run order; Y-axis: standard deviation. (A) QC plot for the first component from LC-(+)ESI-MS data; (B) QC plot for the second component from LC-(+)ESI-MS data; (C) QC plot for the first component from LC-(ESI)-MS data; (D) QC plot for the second component from LC-(ESI)-MS data.**

**Table S1. Information of potential biomarkers after SLG intervention**

| **Name** | **Formula** | **Calculated m/z** | **Observed m/z** | **PPM(Ereer)** |
| --- | --- | --- | --- | --- |
| Tryptophan | C_11_H_12_N_2_O_2_ | 205.09770 | 205.09721 | -2.39 |
| Glutamate | C_5_H_9_NO_4_ | 148.06042 | 146.06021 | 1.28 |
| Serine | C_3_H_7_NO_3_ | 104.10000 | 104.09981 | -1.83 |
| Methionine | C_5_H_11_NO_2_S | 150.05888 | 150.05830 | -3.87 |
| Ornithine | C_5_H_12_N_2_O_2_ | 133.09770 | 133.09920 | 3.76 |
| PA 16:0 | C_35_H_69_O_8_P | 647.46571 | 647.46592 | 2.29 |
| PC 22:5 | C_48_H_84_NO_8_P | 882.60311 | 882.60360 | 2.04 |
| PC 14:0 | C_37_H_69_O_8_P | 659.91860 | 659.91891 | 0.47 |
| Propionylcarnitine | C_10_H_20_ClNO_4_ | 218.13868 | 218.13819 | -2.25 |
| palmitic acid | C_19_H_38_O_4_ | 257.24811 | 257.24870 | 0.78 |
| Acetyl spermidine | C_9_H_21_N_3_O | 188.17629 | 188.17684 | 2.92 |
| sphingosine | C_18_H_37_NO_2_ | 302.29025 | 302.2908 | 1.82 |
| 5-HETE | C_20_H_32_O_3_ | 319.22732 | 319.22741 | 3.41 |
| Azelaic acid | C_9_H_16_O_4_ | 188.09704 | 188.09711 | 1.44 |

()
